# Naringin ameliorates high-fat diet-induced hepatotoxicity and dyslipidemia in experimental rat model via modulation of anti-oxidant enzymes, AMPK and SERBP-1c signaling pathways

**DOI:** 10.1016/j.toxrep.2025.102062

**Published:** 2025-05-30

**Authors:** Sweata Sarkar, Sanjib Ghosh, Maharaj Biswas

**Affiliations:** Endocrinology Laboratory, Department of Zoology, University of Kalyani, Kalyani, West Bengal 741235, India

**Keywords:** Hepatotoxicity, Naringin, NAFLD, Oxidative stress, AMPK signaling, Lipid metabolism, HFD

## Abstract

High-fat diet causes elevation of steatosis, dyslipidemia and oxidative stress which eventually leads to hepatic injury in the form of non-alcoholic fatty liver disease (NAFLD). Naringin, a natural flavonoid, having tremendous potentiality including antioxidant, anti-inflammatory, hypolipidemic role. Based on this proposition, we investigated the role of naringin in hepatotoxicity and its possible underlying mechanism caused by high-fat diet for prolonged time. Fifteen Wistar rats were divided into three groups: Group A (CON) received normal diet; Group B (HFD) was administered with high-fat diet for 16 weeks; and Group C (THN) was treated with naringin (100 mg/kg B.W.) for last 6 weeks after induction of obesity. After autopsy, various parameters were studied like gravimetry, serum biochemistry, ROS activity, anti-oxidant enzymes, genes expression (AMPK and SREBP-1C), histochemistry, histopathology and ultrastructure of hepatic tissue. In HFD group, Masson’s trichome stain intensity increased 6.8-folds, indicating the onset of liver fibrosis; ROS generation and lipid peroxidation (TBARS) were significantly (p < 0.01) increased, whereas SOD and CAT were decreased by 36.7 % and 49.7 %, respectively. Furthermore, these parameters were remained normal in THN group. Besides, HFD group displayed extreme elevation in hepatic SREBP-1C expression (147 %) and downregulation of AMPK gene (77 %) compared to control. The ultrastructural study revealed most important and new insight of this study where HFD induced extreme reticule stress in hepatic tissue which was significantly improved by the treatment of naringin. These findings demonstrate that the naringin may be used as a potential therapeutic agent to combat obesity related hyperlipidemia and NAFLD.

## Highlights

• Naringin administration improves liver functioning in HFD-induced hepatotoxicity.
• Naringin attenuates NAFLD condition by reducing ER stress, hepatic fibrosis, histopathological alterations as well as dyslipidemia in HFD-induced obese rat model.
• Naringin exhibits anti-oxidant, anti-steatosis property which reduces the oxidative stress in NAFLD rat model.
• Naringin downregulates SREBP-1C gene which inhibits lipogenesis alongside upregulates AMPK gene expression and maintaining energy homeostasis in HFD-induced obese rat.

## Graphical Abstract

**Figure:**
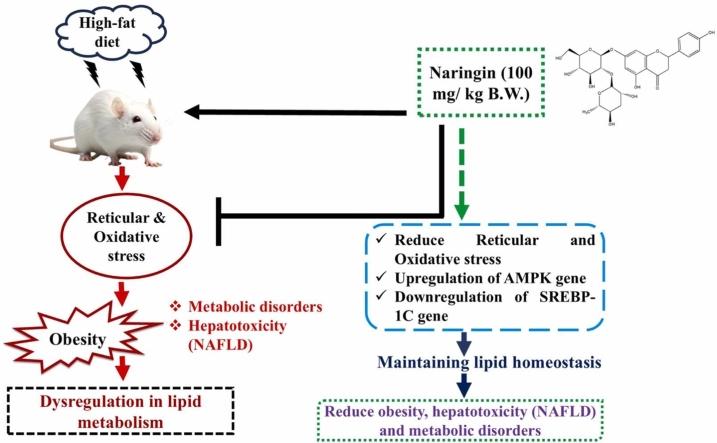

## 1 Introduction

Globally obesity is one of the leading causes of morbidity. WHO has declared obesity as global pandemic. The 2024 WHO report indicates that 890 million individuals were classified as obese out of 2.5 billion overweight adults [1], [2]. The primary etiology of this chronic disease is the consumption of a high-carbohydrate or high-fat diet [3], [4], which ultimately results in dyslipidemia and other metabolic disorders in approximately 60–70 % of obese individuals worldwide [5], [6][5,6]. Obesity influences fat deposition in several organs, including adipose tissue, liver, blood vessels, and skeletal muscles [7] and increases the risk of obesity-linked comorbidities among the obese individuals [8]. The underlying causes of obesity is oxidative stress, which stimulates differentiation and growth of adipocytes [9]. Obesity is associated with metabolic disorders, including cardiovascular diseases (CVDs), hypertension, insulin resistance (IR), along with pulmonary, reproductive and endocrine gland dysfunction [10], [11], [12], [13], [14], [15]. Furthermore, obesity has led to the most underestimated slowly progressing lethal condition, namely non-alcoholic fatty liver disease or NAFLD [16].

HFD contributes to the progression of NAFLD, primarily influenced by the abnormal accumulation of triglycerides as lipid droplets within hepatic tissue, resulting in steatosis. This condition can progress to cirrhosis and hepatocellular carcinoma, indicating hepatotoxicity, as a result it leads to mortality in the NAFLD individuals [17], [18], [19], [20], [21]. The epidemiological data indicates that three-quarters of individuals with obesity are linked to NAFLD [22]. A prior study indicated that approximately 25 % of the overall population in the USA is affected by NAFLD [23]. In western nations, the present situation indicates that liver transplantation ranks as the second most common cause of liver failure attributed to NAFLD [24].

Oxidative stress is the main culprit that causes NAFLD in HFD consuming individuals. HFD is associated with the imbalances in pro-oxidant and anti-oxidant species [25]. This imbalance heightened expression of NADPH oxidase, which further increased activity of reactive oxygen species (ROS) and declined the levels of anti-oxidant enzymes like Superoxide dismutase (SOD) and catalase (CAT) [26], [27]. The generation of ROS resulted in increased lipid peroxidation, protein carbonylation, and mitochondrial DNA damage [28], [29]. Another potential factor for obesity is reticule stress which plays a crucial role in protein folding and lipid metabolism [30]. Impaired endoplasmic reticulum (ER) stress plays a significant role in the progression of NAFLD. The endoplasmic reticulum (ER) is essential for lipid production and processing, as well as metabolism, and is critical for maintaining lipid homeostasis in the liver [31]. Lipid serves as a stimulatory signal capable of disrupting ER activity. When the accumulation of cytoplasmic lipids surpasses the metabolic threshold of the endoplasmic reticulum, it induces a state of ER stress. Disruption in endoplasmic reticulum (ER) homeostasis, referred to as ER stress, initiates hepatic steatosis and the advancement of Non-alcoholic Steatohepatitis (NASH) [32]. Endoplasmic reticulum stress is associated with metabolic processes, contributing to obesity-related issues like insulin resistance and type 2 diabetes [33]. Intracellular stress cascades triggered by endoplasmic reticulum stress could play a role in insulin resistance, inflammation, heightened mortality of hepatocytes, and the development of NASH/NAFLD [34], [35]. Endoplasmic reticulum (ER) stress triggers the activation of the unfolded protein response (UPR), mainly driven by three transmembrane proteins: activating transcription factor 6 (ATF6), protein kinase-like ER kinase (PERK), and inositol-requiring enzyme 1 alpha (IRE1α). Under stress conditions, glucose-regulated protein 78 (GRP78) separates from the transmembrane proteins, leading to an increase in GRP78 expression by inhibiting protein synthesis [36], [37].

Additionally, NAFLD and obesity are inter-linked and gives rise to abnormal lipid metabolism that subsequently promotes hyperlipidemia [38]. This condition is associated with elevated serum total cholesterol (TC) levels, low-density lipoprotein cholesterol (LDL-C), and triglycerides (TG), while high-density lipoprotein cholesterol (HDL-C) levels are decreased [39]. The levels of plasma apolipoprotein (apo) B often exhibit an increase, which can be partially linked to the overproduction of lipoproteins containing apo B in the liver, resulting in changes in lipid metabolism [40]. The buildup of lipids in the vessels caused damage to the endothelium, ultimately resulting in the formation of atherosclerotic plaques, which can progress to acute cardiovascular diseases [41]. The oxidation of LDL serves as the catalyst for the atherogenic process. The presence of lipooxidation products can induce oxidative modifications in LDL particles, leading to the degradation of apolipoprotein B (apo B), which is crucial in oxidative stress and contributes to foam cell formation [42] that leads to inflammatory response [43]. Conversely, a crucial element in the de novo lipogenesis (DNL) pathway is sterol regulatory binding protein 1 (SREBP-1C), a transcription factor that plays a significant role in lipid synthesis by regulating acetyl CoA carboxylase (ACC) and fatty acid synthase (FAS), which are the rate-limiting enzymes [44], [45]. Overexpression of the SREBP-1C gene impaired biogenesis of cholesterol, triglycerides and fatty acids [46]. The SREBP-1C gene expression bidirectionally associated with multiple cellular processes like ER stress, ROS generation, apoptosis and inhibits autophagy, contributing to the pathogenesis of NASH/NAFLD [47]. Additionally, a significant factor, AMPK, known as the energy sensor protein, has recently garnered increased interest due to its role in inhibiting lipogenesis and functioning as a negative regulator of SREBP-1C. Its activation leads to lipid degradation, autophagy, enhanced glucose uptake, and a reduction in oxidative stress through the downregulation of lipogenesis genes [48], [49]. Previous studies have shown that phosphorylation of the AMPK gene enhanced autophagy and fatty acid oxidation *via* inhibiting acetyl-CoA carboxylase (ACC) and fatty acid synthase (FAS) expressions [50], [51]. The modulations of this gene result in increased lipid accumulation and contribute to NAFLD conditions as a result of extended periods of high-fat diet consumption [52]. The interplay between these two genes (SREBP-1C and AMPK) may be a promising therapy to treat HFD-induced hepatotoxicity.

Various therapies and treatments are conventionally used and practised to control obesity and obesity-linked disorders like hyperlipidemia through managing different mechanisms and pathways like promoting lipolysis, facilitating weight loss through gut hormone regulation, managing lipid digestion and absorption, controlling appetite, and implementing behavioural modifications [53], [54]. There are some FDA-approved drugs that are widely used as medications for the treatment of various metabolic disorders, such as orlistat, atorvastatin, liraglutide, metformin, sibutramine, phentermine, bupropion, and fenofibrate, which have adequate clinical effectuality but with severe side effects [55], [56], [57], [58]. Pharmaceuticals that focus on lipid mobilization, implementation, and the attenuation of nutritional absorption represent a critical therapeutic strategy [53], but these drugs possess several side effects that are harmful for human health. So, a new strategy should be implemented in this field.

Phytoconstituents are more effective with less toxic effects, widely used as traditional medicine since ancient times and play a crucial role in obesity-related dyslipidemia, hepatotoxicity, oxidative stress, lipolysis of lipid, and appetite management. Flavonoids are plant secondary metabolites widely spread in citrus fruits, vegetables, seeds, and nuts showing antioxidant properties via reducing lipid peroxidation levels. Naringin is a flavone glycoside found in grapefruit; naringin, when consumed orally, it hydrolyzes into its aglycone naringenin [59]. Naringin is a potent flavonoid with enormous therapeutic efficacy: antioxidant, anti-obesity, hypolipidemic, and hepatoprotective properties that prevent lipid oxidation, oxidative stress, inhibit lipoxygenase, and LDL-C oxidation, which in turn impairs the differentiation of adipocytes, inhibits lipid accumulation, promotes lipolysis, and mitigates inflammation [60], [61], [62]. Aside, naringin serves as a therapeutic potential agent in the cardioprotective [63], [64], [65], hepatoprotective [59], [66], [67], anti-cancer [68], [69], anti-inflammatory [70], metabolic syndrome [71], [72], [73], and anti-bone regeneration fields [74].

Nowadays high-fat diet (HFD) ingestion is increasing day by day with our unhealthy dietary practice, and for prolonged intake eventually leads to the hepatotoxicity by induction of NASH/ NAFLD. Therefore, we like to bring attention to this global epidemic non-communicable disease and highlight the promising treatment with the bioactive flavonoid naringin. The main objective of the current study is to investigate the efficacy of naringin as a potential hepatoprotective and hypolipidemic agent through antioxidant defense as well as SREBP-1C and AMPK signaling pathways in liver injury caused by chronic high-fat diet feeding in Wistar rats.

## 2 Materials and methodology

### 2.1 Chemicals and drugs

Naringin (MW. 580.53 g/mol) purity ≤ 95 %, DCFDA were obtained from Sigma-Aldrich Corporation (St. Louis, MO, USA). Thiobarbituric acid and paraformaldehyde were purchased from LOBA ChemiePvt. Ltd. Haematoxylin, sudan III, sudan IV, riboflavin, L-methionine and all other chemicals were purchased from SRL Pvt. Ltd. in analytical grade.

### 2.2 Animals

Male Wistar rats weighing about 80–100 g were used to carry out the whole experiment. Animals were kept in a polycarbonate cage with a top grill. Rat standard pellet or formulated high-fat diet and water were given *ad libitum.* All animals were acclimatized for one week at 23 ± 2°C temperature with 50–60 % relative humidity and a 12 h light and dark cycle. Animal ethical permission was in accordance with the Institutional Animal Ethical Committee of the University of Kalyani, Kalyani, Nadia, West Bengal, India (Reg. no. 892/GO/Re/S/01/CPCSEA).

### 2.3 Induction of obesity

The high-fat diet contains 40 % fat, 43 % carbohydrates, and 17 % proteins. The ingredients (g/100 g) are powdered rat feed 68.0 g, corn oil 6.0 g, butter 6.0 g, milk powder 20.0 g, and egg. The high-fat diet was given to group II for consecutive days during the entire period of the experiment (16 weeks). Normal rat chow was given to Group I (control) during the tenure of the experiment. Naringin was orally administered at a dose of 100 mg/kg BW from the 10th to the 16th week of the experiment.

### 2.4 Experimental design

15 male Wistar rats were allocated into 3 groups; each group contained 5 animals.

**Group A (CON):** Control and fed with normal rat pellets for 16 weeks.

**Group B (HFD):** High-fat diet (HFD) group administered with a 40 % fat diet for 16 weeks.

**Group C (THN):** Fed with HFD for 10 weeks, and naringin was delivered at a dose of 100 mg/kg body weight orally for the last 6 weeks continuously.

### 2.5 Blood and tissue collection

Following the intervention period of 16 weeks, rats were anaesthetized using a dose of 10 mg/kg xylazine and 80 mg/kg ketamine intraperitoneally and then sacrificed. Blood was immediately collected from heart and centrifuged at 3000 rpm for 10 min for separation of serum, which was aliquoted a centrifuge tube and stored at −20ºC for further analysis. The liver and adipose tissue were collected, and measurements of weight were performed. Organs were stored at −80ºC for measurement of other assays.

### 2.6 Morphometric analysis

The body weight of each rat was evaluated regularly. Upon concluding the experiment, final body weight was assessed, and length was measured with a measuring tape to compute BMI. For the morphometric investigation, final body weight, weight of liver and adipose tissue, liver and visceral adiposity index, and Lee's index were determined using the data.

### 2.7 Serum lipid profile assessment

All biochemical parameters were measured using the biochemistry analyser “Prietest Easy Lab”, Robonik India Pvt. Ltd., Mumbai, India. Serum total cholesterol (TC, lot no. 221003Robonik), total triglycerides (TG, lot no. 212902Robonik), high density lipoprotein (HDL, lot no. 22DX013 Reckon), serum low-density lipoprotein (LDL), and very-low density lipoprotein (VLDL) were calculated using Friedewald’s equation [75].

VLDL-C (mg/dl) = TG/5; LDL-C (mg/dl) = TC-(HDL-C+VLDL)

### 2.8 Assay of serum liver toxicity biomarkers and digestive enzymes

liver toxicity biomarkers like alanine aminotransferase (ALT, lot no. 221503 Robonik), aspartate aminotransferase (AST, lot no. 221602 Robonik), and alkaline phosphatase (ALP, lot no. 220302 Robonik) were estimated using enzymatic assay kits. The lipase assay kit (LIP210801 Delta) and the amylase assay kit (AM202308Diatek) were used to measure serum lipase and amylase activity.

### 2.9 Histopathological studies of hepatic tissue

The livers of the sacrificed rats were dissected out and fixed with 10 % neutral buffer formalin (NBF) for 24–48 h, which were dehydrated using graded alcohol and prepared a paraffin block. 4–5 µm tissue sections were fixed in a glass slide with Mayer’s albumin and stained with haematoxylin and eosin (H-E). For each group, five slides were assessed for histological alterations under the Carl Zeiss Primo Star microscope, and the NAFLD score was assessed using three criteria, *i.e.*, lobular inflammation, steatosis and hepatocyte ballooning [76]. For collagen distribution throughout the liver tissue, Masson’s trichrome stain was performed and analyzed further for hepatic fibrosis assessment [77] under the microscope (Carl Zeiss, Primo Star).

### 2.10 Histochemical studies of hepatic tissue

Lipid accumulation in the hepatic tissue was estimated using histochemical staining of lipids from fresh hepatic tissues. Liver tissues were fixed at −20°C, and the section was cut by cryostat (Reichert-Jung) and stained with the Sudan Black B method [78], which was further investigated under microscope for any kind of alterations.

### 2.11 Assay of ROS generation in hepatic tissue by DCFDA method

The ROS generation in liver tissue was determined using 2’, 7’-dichlorofluorescin diacetates (DCFDA) staining [79]. The livers from the sacrificed animals were excised and homogenized with PBS and centrifuged at 3000 g for 10 min and incubated for 15 mins with DCFDA at room temperature, and quantification was done by spectrofluorometer with an excitation of 485 nm and an emission of 530 nm.

### 2.12 Measurement of antioxidant defense markers

After completion of the experiment, the liver was dissected out, washed with PBS, blotted, and weighed. After that homogenate solution was prepared, further analysis was done with the supernatant. Liver oxidative stress was determined by following enzymatic assays like thiobarbituric acid reactive substances (TBARS), superoxide dismutase (SOD), and catalase (CAT) assay [80], [81], [82]. Total protein was estimated by the Lowry method [83].

### 2.13 qRT-PCR analysis

Total RNA was extracted from the liver tissues utilizing PureZOL (BioRAD, California, USA). We quantified the RNA using a UV spectrophotometer and synthesised it using the QuantiTect Reverse Transcription Kit (BioRAD, California, USA). Real-time polymerase chain reaction (RT-PCR) was conducted utilizing a SYBR green supermix RT-PCR kit (BioRAD, California, USA), and primers were designed (see Table 2). The gene expression levels of AMPK and SREBP-1C were analyzed, and GAPDH was used as an internal control.

**Table 1:** Changes in morphometric study in between control and treatment groups. Data are presented as Mean ± SEM, N = 5; the mean difference is significant at: *^a^p < 0.05, * *^a^p < 0.01 CON vs HFD; HFD vs NAR, *^b^p < 0.05, * *^b^p < 0.01 level.

**Parameters**	**CON**	**HFD**	**THN**
**Food intake (g/day)**	19.06 ± 0.90	15.59 ± 0.83^#a^	19.20 ± 0.53^#a, #b^
**Body weight gain (g)**	73.23 ± 1.66	137.88 ± 3.79^**a^	81.59 ± 2.52^*a,**b^
**BMI (g/cm**^**2**^**)**	0.46 ± 0.01	0.71 ± 0.01^**a^	0.47 ± 0.02^#a, **b^
**Lees’s index (g/cm)**	292.21 ± 1.86	335.49 ± 2.33^**a^	293.31 ± 3.09^#a, **b^
**Adipose tissue weight (g)**	5.83 ± 0.13	13.74 ± 0.44^**a^	6.69 ± 0.17^#a, **b^
**Liver weight (g)**	4.60 ± 0.18	10.23 ± 0.27^**a^	5.72 ± 0.40 *^a, **b^

**Table 2:** Forward and reverse primer of the following genes.

GAPDH F	CCTCGTCTCATAGACAAGATGGT
GAPDH R	GGGTAGAGTCATACTGGAACATG
SREBP F	TCCTCACTCCCTCTGATGCT
SREBP R	TTGCGATGTCTCCAGAAGTG
AMPK F	CATTTGTGCAAGGCCCCTAGT
AMPK R	GACTGTTGGTATCTGCCTGTTTCC

### 2.14 Ultrastructural study for analyzing ER stress and mitochondrial dysfunction

To prepare the sample for transmission electron microscopy, liver tissue was extracted from each rat and sectioned into 1 mm³ pieces. The liver sample was fixed using Karnovsky’s fixative and stored at 4°C. Subsequently, rinse the sample using cold cocodylate buffer to eliminate any surplus fixative. Following that, immerse the specimen in 1 % osmium tetraoxide and proceed to dehydrate the tissue using graded alcohol to eliminate any water content. Subsequently, embedding the tissue in fresh spurr’s resin and placing it in a hot oven for polymerization over a period of 24 hours. Carefully trim the block and cut the ultrathin sections using an ultramicrotome, then stain with uranyl acetate for further examination under the Talos transmission electron microscope (ThermoScientific, U.S.).

### 2.15 Statistical analysis

All the results were depicted as mean ± SEM for 3 groups containing five animals each. All the data of the respective groups were statistically evaluated using GraphPad Prism 9.0 software. Statistical analysis was done using one-way ANOVA, and Tukey’s test, at p < 0.05 and p < 0.01 significance levels. Data were validated using the Shapiro-Wilk test and the Box plot method.

## 3 Results

### 3.1 Effects of Naringin on body weight, BMI, relative organ weight, liver index, Lee’s index and food intake

Table 1 illustrates the alterations in caloric consumption, body weight gain, body mass index (BMI), adipose tissue mass, adiposity index, Lee's index, hepatic mass, and liver index after 16 weeks of treatment. The HFD group exhibited a significant increase in fat mass, BMI, body weight gain, adiposity index, and Lees’s index (p < 0.01), suggesting the emergence of obesity, relative to rats fed on a normal diet. The administration of naringin (100 mg/kg B.W.) led to a significant (p < 0.01) decrease in the weight gain associated with high-fat diet consumption. No notable change in food consumption was seen across all groups.

### 3.2 Effects of Naringin on Serum lipid profile

**Fig. 1:**
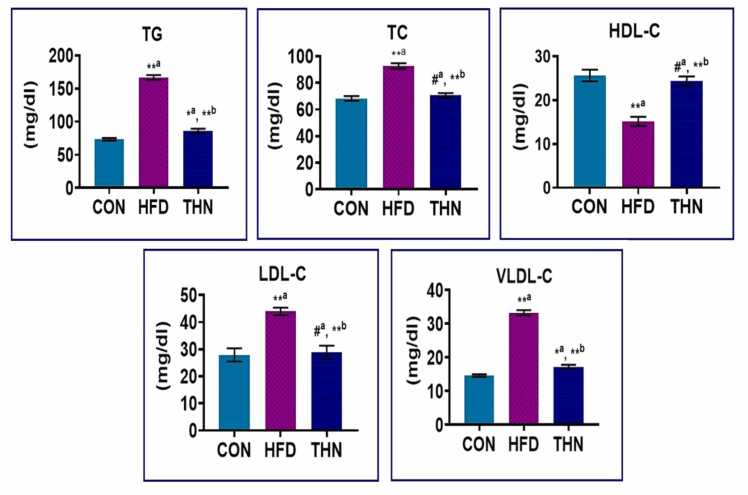
Graphical representation of serum lipid profile in control and treatment groups. The results are expressed as Mean ± SEM, significance levels were analysed by using one-way ANOVA followed by Tukey test. N = 5; Comparison was done between CON with HFD and HFD with THN. Statistical significance *^a^p < 0.05, * *^a^p < 0.01 CON vs HFD; HFD vs NAR, *^b^p < 0.05, * *^b^p < 0.01 level.

Fig. 1 highlights the blood lipid profile, encompassing serum triglycerides (TG), total cholesterol (TC), high-density lipoprotein (HDL), very low-density lipoprotein (VLDL), and low-density lipoprotein (LDL) levels. The findings indicates that the HFD group had dramatically higher levels of TC, TG, and VLDL (p < 0.01), as well as an elevated LDL level (p < 0.05) in comparison to the CON group. A significant reduction (p < 0.01) in serum HDL levels was also noted in the HFD group. The THN group demonstrated a significant decline in TG, TC, LDL, and VLDL levels (p < 0.01) while, exhibited a significant incline (p < 0.01) in high-density lipoprotein (HDL) levels.

### 3.3 Effects of Naringin on liver toxicity enzymes

**Fig. 2:**
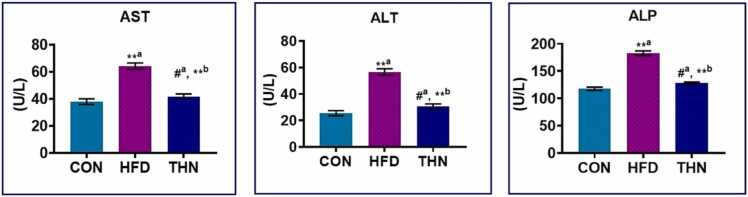
Alteration of liver biomarker in different groups. Values are expressed as Mean ± SEM, N = 5; the mean difference is significant at: *^a^p < 0.05, * *^a^p < 0.01; CON vs HFD; HFD vs NAR, *^b^p < 0.05, * *^b^p < 0.01. Significance levels were analysed by using one-way ANOVA and Tukey’s test.

Fig. 2 illustrates that the HFD group exhibited a significant increase (p < 0.01) in serum liver function markers such as ALP, AST, and ALT when compared to the control group, indicating hepatotoxicity. The ingestion of naringin by HFD-fed animals over the past 6 weeks led to a notable (p < 0.01) enhancement in the attenuation of liver function biomarkers affected by HFD. The concurrent administration of naringin at a dosage of 100 mg/kg BW showed a reduced effectiveness in mitigating the changes induced by a high-fat diet. The THN group exhibited no statistically significant differences when compared to the CON group.

### 3.4 Effects of Naringin on serum lipase and amylase

**Fig. 3:**
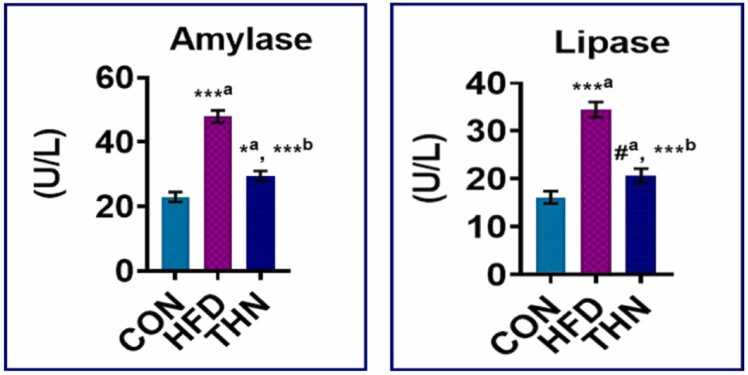
Graphical presentation of different serum digestive enzymes. Data are expressed as Mean ± SEM, N = 5; the mean difference is significant at: *^a^p < 0.05, * *^a^p < 0.01; CON vs HFD; HFD vs NAR, *^b^p < 0.05, * *^b^p < 0.01. Significance levels were analysed by using one-way ANOVA and Tukey’s test.

Moreover, serum amylase and lipase activity (Fig. 3.) substantially increased (p < 0.01) in the high-fat diet group after 16 weeks of treatment compared to the control group. In the THN group, administration of naringin resulted in a significant reduction in blood amylase and lipase levels (p < 0.05) compared to the HFD group, despite no significant change observed when compared to the control group.

### 3.5 Effects of Naringin on histopathology of hepatic tissue

**Fig. 4:**
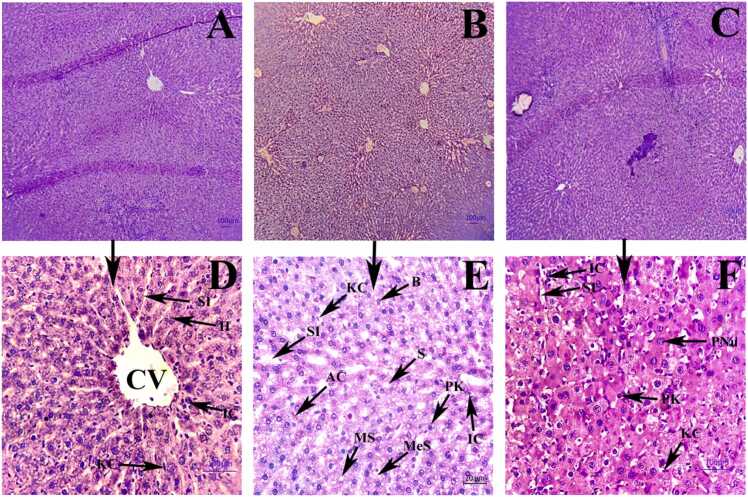
Photomicrograph of histological alterations in liver (100x and 400x) in different groups. A (CON), B (HFD) and C (THN) were examined under 100x magnification. Whereas, D (CON), E (HFD), F (THN) were examined under 400x magnification. HFD group showed Inflammatory cells (IC), Ballooning cell structure (b), steatosis (S), massive dilation of sinusoids (SD) than other two groups.

The results of the H-E staining (Fig. 4) of liver tissue across various groups showing no discernible pathogenic changes were seen in the CON group, which displayed typical anatomy featuring sinusoids and a central vein. Compared to the CON group, in the HFD group exhibited noticeably increase of the NAFLD score. Degenerated hepatocytes exhibiting necrosis, ballooning cells with Mallory-Denk bodies (B), acidophil bodies (AC), micro and mega vesicular steatosis (MeS), massive inflammatory cell infiltration (IC), indistinct lobular structure, sinusoidal dilatation (SD), a large number of Kupffer cells (KC) and degenerated hepatic cell mass were exhibited in the HFD group. However, naringin treatment changed these hepatocytic lesions in comparison to the HFD group. Histology examination revealed that supplementation with naringin reversed the hepatic injury caused by HFD treatment. The sinusoids showed recovery signs, polynucleated hepatocytes, fewer Kupffer cells, pyknotic nuclei, and a decrease in the number of ballooning cells. The histology section also showed a decline in the number of apoptotic cells, inflammatory cells, and microsteatosis. This result indicates that naringin administration mitigated hepatotoxicity-associated histopathological architecture alterations induced by high-fat diet treatment.

### 3.6 Effects of Naringin on liver fibrosis

**Fig. 5:**
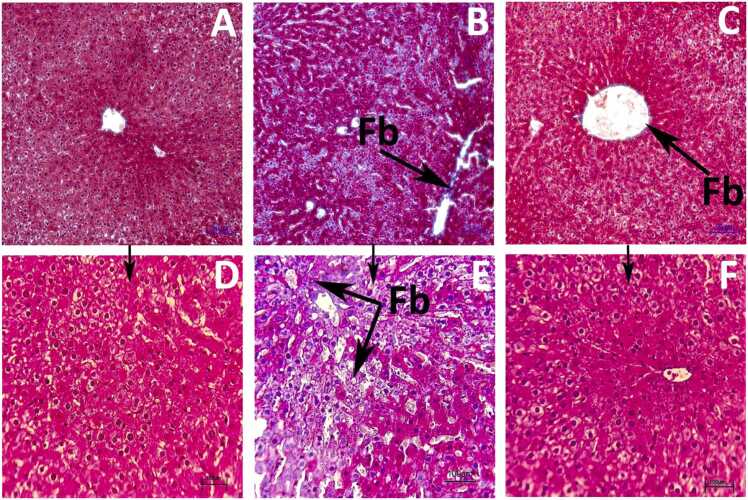
Collagen III distribution in liver tissue indicating the liver fibrosis grade. Magnification 200x (A, B, and C); 400x (D, E, and F). A, D (CON) showing no fibrosis in hepatic tissue, B, E (HFD) showing massive level of fibrosis whereas C, F (THN) showing reduction in collagen tissue distribution. Tissue sections were stained with Masson’s Trichome staining.

Liver fibrosis is one of the characteristics of NAFLD/NASH. This assessment was done by Masson’s trichrome staining. Masson’s trichrome (MT) staining (Fig. 5) was used for the detection of liver fibrosis. In CON group no such fibrous condition was observed. In the HFD group fibrous network was exhibited, which bridged from the portal vein to the central vein or another portal vein. The naringin treatment group showed a reduction of fibrous condition.

### 3.7 Liver fibrosis and NAFLD scoring in hepatic tissue

**Fig. 6:**
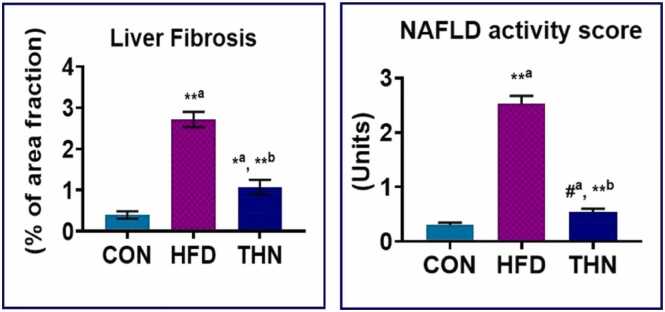
Graphical presentation of liver fibrosis score and NAFLD activity score in hepatic tissue. Values are depicted as Mean ± SEM, N = 5; the mean difference is significant at: *^a^p < 0.05, * *^a^p < 0.01; CON vs HFD; HFD vs NAR, *^b^p < 0.05, * *^b^p < 0.01. Significance levels were analysed using One-way ANOVA and Tukey’s test.

Fig. 6. showed that in the HFD group, liver fibrosis was extremely elevated (p < 0.01). Whereas, the THN group showed a significant decline (p < 0.01) in compare to the HFD group. In comparison with CON group, THN group showed significant (p < 0.05) increased. NAFLD activity was assessed from histological examination. The NAFLD activity score was remarkably (p < 0.01) elevated in the HFD treatment compared to the CON group. THN group showed significant (p < 0.01) decreases compared to the HFD group. The THN group remains unchanged when compared to the CON group.

### 3.8 Effects of Naringin on lipid accumulation in hepatic tissue

**Fig. 7:**
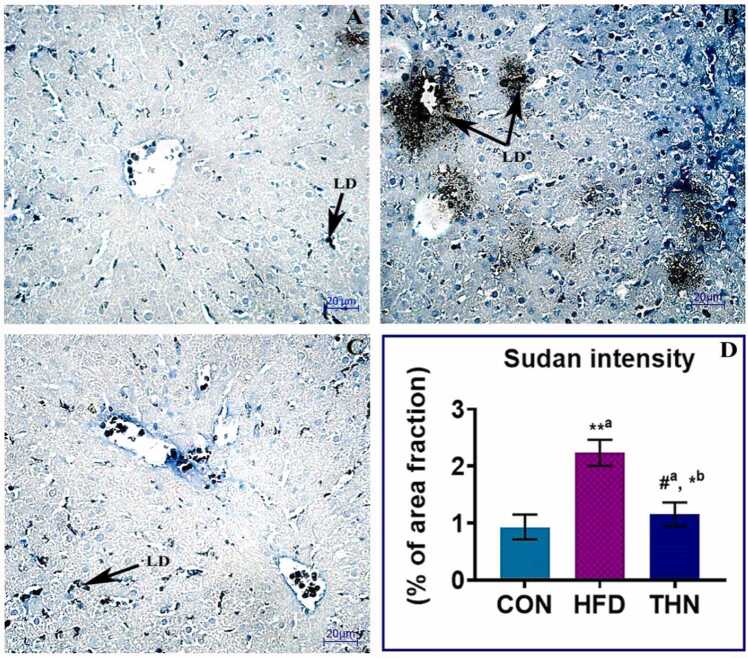
Photomicrograph of Sudan Black B staining in different groups. Magnification 400x. Group B (HFD) showing excessive fat accumulation in hepatic tissue in the form of lipid droplet than Group A (CON) and group C (THN). Sudan Black B intensity was statistically analysed using One-way ANOVA. Values are depicted as Mean ± SEM, N = 5; the mean difference is significant at: *^a^p < 0.05, * *^a^p < 0.01 CON vs HFD; HFD vs NAR, *^b^p < 0.05, * *^b^p < 0.01level.

Sudan Black B staining (Fig. 7) for lipid distribution was performed in hepatic tissue. Expression of lipid accumulation was significantly (p < 0.01) much higher in concentration in the HFD group (B) in comparison with the control group (A). In this regard, group C (THN) significantly (p < 0.05) mitigated the intracellular hepatic lipid deposition near to control after 6 weeks of treatment of obesity introduced in comparison to the HFD group. No changes occurred in the THN group when compared to the CON group.

### 3.9 Effects of Naringin on oxidative stress

**Fig. 8:**
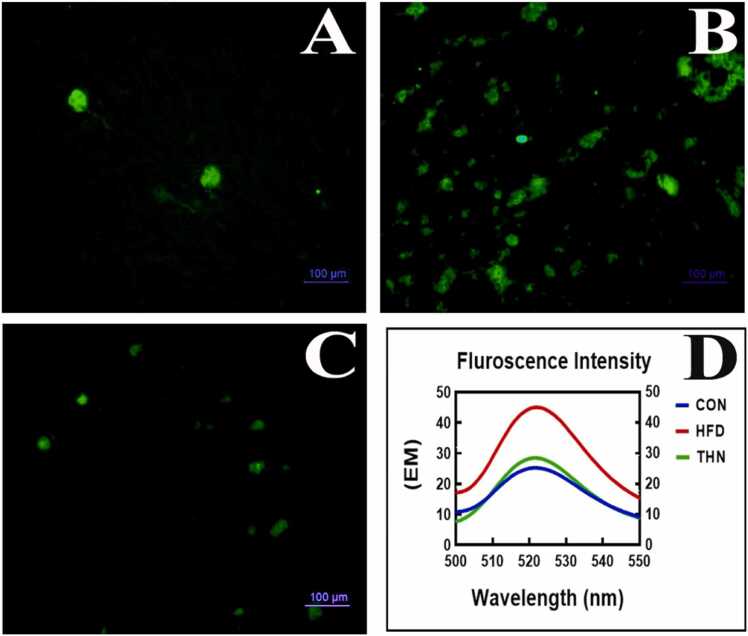
Detection of oxidative stress by DCFDA staining and spectrofluorimetric study for quantification of ROS generation in different groups. A (CON), B (HFD), C (THN) were examined under 400x magnification with DCFDA staining. D represented the graphical representation of quantitative study of ROS generation by spectrofluorometer. In both cases HFD group showed increased amount of ROS generation in hepatic tissue.

The DPPH radical scavenging experiment was performed for validation of ROS generation. In hepatic tissue (Fig. 8), the HFD group displayed strong intensity, and the same result was found in spectrofluorometric investigation. Consistent high-fat diet consumption caused ROS generation. The qualitative and quantitative study, indicating hepatic toxicity or liver injury in HFD group. After obesity induction, naringin (100 mg/kg B.W.) was administered for 6 weeks, showing ameliorative benefits and reducing oxidative stress.

### 3.10 Effects of Naringin on antioxidant defence in liver

**Fig. 9:**
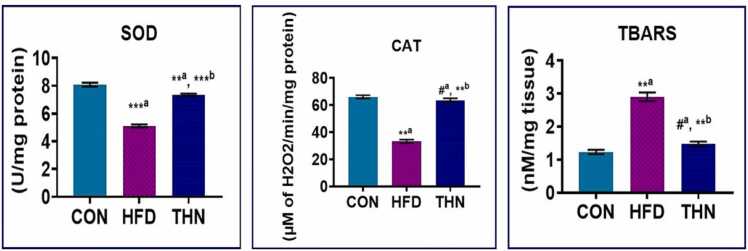
Values are presented as Mean ± SEM, N = 5; the mean difference is significant at: *^a^p < 0.05, * *^a^p < 0.01, CON vs HFD; HFD vs NAR, *^b^p < 0.05, * *^b^p < 0.01 level. Antioxidant status was assessed by SOD, CAT (A) and lipid peroxidation (B) using one-way ANOVA.

The HFD group displayed a more vigorous elevation (p < 0.01) in lipid peroxidation level in the hepatic tissue than the CON group. Simultaneously, catalase and SOD values significantly reduced (p < 0.01) when compare to the CON group. Naringin exhibited a remarkable hepatoprotective role via reducing oxidative stress. In the THN group SOD, CAT level (Fig. 9) significantly (p < 0.01) increased, whereas TBARS level significantly (p < 0.01) decreased in comparison with the HFD group.

### 3.11 Effects of Naringin on *AMPK* and *SREBP-1C* gene expression

**Fig. 10:**
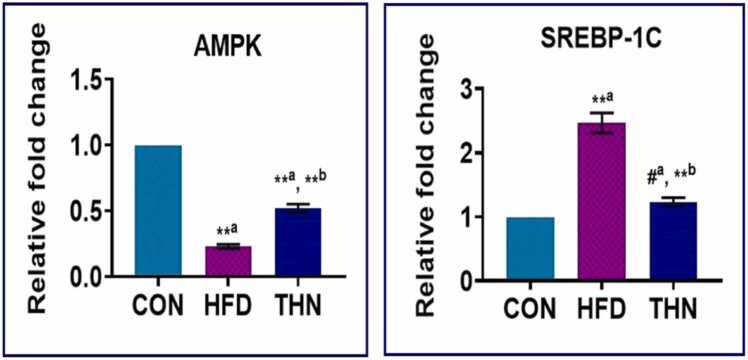
Graphical representation of SREBP-1C and AMPK gene expression in different groups. Values are presented as Mean ± SEM, N = 5; the mean difference is significant at: *^a^p < 0.05, * *^a^p < 0.01, CON vs HFD; HFD vs NAR, *^b^p < 0.05, * *^b^p < 0.01 level.

Fig. 10 Clearly depicted that naringin downregulates (p < 0.01) SREBP-1C expression in comparison with the HFD group. Whereas, as compared to the control group, the HFD-fed rat showed a significantly increased value of SREBP-1C expression, but the naringin group showed no such significant change when compared to the control group. AMPK is a crucial protein in the maintenance of energy homeostasis. In comparison with the CON group, the HFD group showed significant (p < 0.01) downregulation of the AMPK expression, but in the THN group, upregulation of AMPK activation (p < 0.01) occurred in comparison with the HFD group. Supplementation with naringin showed increased AMPK expression but comparatively lower than the CON group.

### 3.12 Ultrastructural changes in hepatic tissue by transmission electron microscope

**Fig. 11:**
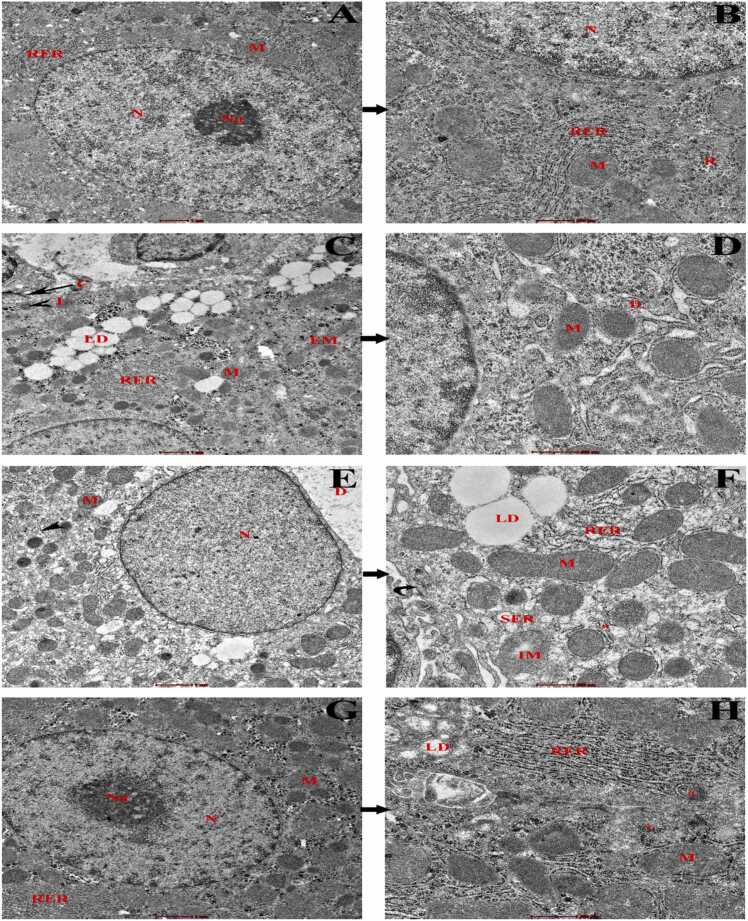
Transmission electron micrographs of rat liver sections. Control rats (**A, B**) showing rounded euchromatin nuclei (N) with prompt nuclear membrane and nucleolus (Nu). Normal architecture of double membranous mitochondria (M) encompassed with the cisternae of rough endoplasmic reticulum (RER) as well as ribosomes is distributed throughout the cytoplasm. The HFD group (**C, D, E, F**) shows various sizes of lipid droplets (LD) throughout the hepatocytes, shrinkage of the nuclear membrane, small nuclei (N) dominated by heterochromatin, and mitochondria (M) in different sizes; some elongated mitochondria (EM), and some mitochondria are fused with lipid droplets (IM). Hepatocytes became vacuolated and also showed swollen rough endoplasmic reticulum (RER), dilated RER (D), and fragmented RER (*). Bile canaliculi show distorted microvilli (curved arrow), presence of electron-dense lysosomes (arrowhead), and collagen deposition (C). The THN group (**G, H**) shows a prominent nucleolus (Nu) with euchromatin-encompassed nuclei, a well-defined nuclear membrane, presence of autophagosome (#), a distribution of continuous RER throughout the cytoplasm, and some fragmented RER are also present. Normal structure of mitochondria, presence of lipid droplets, but the amount and size are reduced.

Electron microscopy (Fig. 11) was conducted at the ultrastructural level to evaluate mitochondrial (M) dysfunction and endoplasmic reticulum (ER) stress in the hepatic cells. Control rats displayed normal rounded-shaped nuclei (N) with prominent nucleoli (Nu), continuous distribution of cytoplasm as well as other organelles, and distinct mitochondrial (M) structure. Presence of orderly distributed ER attached with ribosomes, no dilation or fragmentation of ER. In the HFD group, hepatocytes showed a small nucleus and were predominantly packed with heterochromatin, had a shrunken nuclear membrane, and were degenerative in nature. Mitochondria were numerous in number, and alterations occurred in the structure. Some of them showed elongation, some were fused with lipid droplets showing impairment, including edema, and an electron-dense matrix was also detected in the livers of rats subjected to a high-fat diet. The cytoplasm of the hepatocytes was filled with abundant lipid droplets, dilated ER, fragmented ER, swelling of ER, and a decrease in the number of ER in the cytoplasm. Cytoplasm was discontinuous in nature, and distortion of bile canaliculi was also observed. The ultrastructure of hepatocytes in the THN group showed rounded nuclei with prominent nucleolus, mitochondrial size was normalized, continuous distribution of RER, some fragmented ER, and reduction of lipid droplets in size and number. Transmission electron microscopy of hepatocyte confirmed that treatment with naringin increased the number of autophagosome than HFD group.

## 4 Discussion

The current study demonstrated that ingestion of a high-fat diet introduces obesity-associated dyslipidemia, NASH/NAFLD. Previous findings also suggested the same that intake of HFD for 4 weeks resulted increase of muscle triglycerides which further infleunces visceral obesity and insulin resistance [84]. Excessive calorie consumption leading to the development of visceral fat, which disrupts adipocytes and generates free radicals causing oxidative stress, which in turn results in dyslipidemia, dysregulation of digestive enzymes, elevated serum levels of liver injury markers, and impairs lipid metabolism. A previous study also suggested that oxidative stress is linked to obesity and modifications in lifestyle may alter this phenomenon [85].

Considering the therapeutic potentialities of naringin it was proposed that supplementation of naringin could attenuate the high-fat diet-induced oxidative stress, hepatic injury, and hyperlipidemia via regulating genes of lipid metabolism. HFD also causes elevation of inflammatory markers. The previous report suggested that naringin has a great antioxidant property, which reduces the inflammation by suppressing ROS generation [67]. In the present study, alteration in lipid profile caused by HFD (Fig. 1) resulted in the risk of coronary heart diseases. Treatment with naringin significantly decreases serum TC, TG, LDL-C, and VLDL-C levels, whereas elevating the level of HDL cholesterol. A previous report identified that elevation of HDL-C plays a major role in facilitating the transport of surplus cholesterol to the liver for production of bile [86]. In the present study, supplementation of HFD attributed to the rise of lipid profile in both plasma and liver of experimental rats except HDL-C, not only dyslipidemia but also resulted into hepatotoxicity ultimately leads to NAFLD. Further, the alteration of the lipid profile induced by HFD may be facilitating the activation of lipase enzyme, intestinal fat absorption, and finally lipolysis. Inhibition of lipase causes a reduction in fat digestion as well as delayed fat absorption in the intestine, as supported by previous study [87]. Same as, α-amylase, the inhibition may hinder carbohydrate digestion, which in turn reduces energy intake [88]. Supplementation with naringin exhibited a reduction of the elevated lipase and amylase activity compared to the HFD group. Priscilla et al., (2014) suggested that naringin not only decreased amylase and lipase activity but also reduced the α-glucosidase activity which results into reduction of glucose absorption. This could be a therapeutic approach for treatment of obesity and hyperlipidemia as well as hyperglycemia [89]. In the HFD group, the increase of body weight gain, adiposity index, Lees’s index, fat mass, BMI, energy intake, and other biochemical parameters pointed towards the onset of metabolic disorder (Table 1). Whereas these parameters were notably altered by oral supplementation of naringin at a dose of 100 mg/kg body weight, which influenced the metabolic alterations compared to the HFD group. These pieces of evidences, coupled with the previous report, stated that treatment with naringin resulted in the improvement of liver mitochondrial dysfunction, glucose tolerance, and lipid accumulation, as well as maintaining the liver and heart relative weight [90].

Regarding liver toxicity enzymes, the present study depicted that liver toxicity enzymes such as AST, ALT, and ALP significantly increased in the HFD group (Fig. 2), which indicates damage in the liver or maybe fat accumulation in hepatic tissue, which caused liver steatosis and resulted in the elevation of these markers. Administration of naringin at 100 mg/kg body weight remarkably reduced the ALT, AST, and ALP levels compared to the HFD-treated group. A previous study demonstrated that naringin effectively declined excessive weight gain of the liver alongside liver injury markers like AST and ALT, which denoted that naringin was repairing the damaged hepatic tissue as well as steatosis caused by HFD consumption [67].

Regarding oxidative stress, from the current study, we clearly depicted that naringin significantly declines the lipid peroxidation levels, whereas increased SOD and catalase activity indicates that naringin directly reacts with free radicals and suppresses the ROS generation via free radical scavenging activity (Fig. 9 and Fig. 8). Our findings are in line with the previous report that the HFD-induced model showed an elevation in ROS generation, and the treatment with naringin altered this oxidative stress as well as reduced inflammatory cell infiltration [90].

In the HFD group, histopathological study revealed that the NAFLD activity score and fibrosis score were elevated in comparison to the CON group (Fig. 6). H-E and Masson’s trichrome staining of hepatic tissue (Fig. 4 and Fig. 5) explained that the HFD group showed degenerated structure of liver tissue, dilation of sinusoids, lobular inflammation, condensation of nuclei, excessive presence of Kupffer cells, loss of cytoplasmic mass, and steatosis as well as fibrosis. Supplementation of naringin at a dose of 100 mg/kg BW orally improved all kinds of lesions. Recent studies also established that naringin supplementation strongly showed hypolipidemic effects and mitigated fatty liver condition in mice model [39]. A previous study depicted that naringin administration attenuated gastrointestinal fibrosis by inhibiting ER-stress in male C57BL/6 mice model [91].On the other hand, naringin suppressed the lipid synthesis pathway which eventually leads to inhibition of lipid deposition, dyslipidemia moreover metabolic diseases [92]. In the present study, histochemical study of lipid by Sudan staining (Fig. 7) also validated this reporting.

The Liver is the main compartment of our system, which maintains all the metabolic functions. Lipogenesis of fatty acids and lipids, synthesis of cholesterol and bile production takes place here. So, the ingestion of high-fat diet caused upregulation of lipogenic genes such as SREBP-1C associated with another transcription factor ChREBP regulates de novo lipogenesis process. This gene expression targets FAS and ACC expression, which increases the TG pool [93], [94]. Another important gene AMPK contributing to the maintenance of energy homeostasis not only energy but also oxidative stress. HFD consumption for a long time resulted in the impairment of AMPK genes, which causes dysfunction in glucose uptake and liberation of free radicals, ultimately oxidative stress [95]. After induction of obesity, administration of naringin showed downregulation of these lipogenic gene expressions and inhibited DAGT2 and GPAT-1 expression, which contributed to the reduction in TG synthesis and manifesting decreases in the serum and hepatic TG level. Furthermore, naringin phosphorylates AMPK, which activates AMPK and results in β-oxidation of fatty acids, inhibition of gluconeogenic enzymes, absorption of fat and triggering the Nrf/HO-1 pathway, which inhibits the ROS generation and reduces oxidative stress [96], [97]. Naringin upregulated the AMPK gene expression after 6 weeks of treatment. Our study is also in agreement with the aforementioned studies (Fig. 10).

The ultrastructural examination of the HFD group revealed that the irregular shape of nuclei with chromatin condensation, enlargement of mitochondrial size, and changes in shape, as well as the mitochondrial matrix becoming electron dense (Fig. 11), pointed towards mitochondrial dysfunction, and these findings of the present study are in good agreement with the previous studies of Stacchiotti et al. [98], [99]. Apoptosis-related active substances in the mitochondria contributed to the release of cytochrome c into the cytoplasm. The enlarged mitochondria represent significant morphological alterations associated with mitochondrial dysfunction, which eventually leads to oxidative stress [100]. Electron micrographs were also correlated with the histological and histochemical examination (Fig. 4 & Fig. 7), which revealed an accumulation of different sizes of lipid droplets in the hepatocytes contributed to vacuolated cytoplasm. Stephenson et al. reported that prolonged time ingestion of HFD resulted in hepatic steatosis [101]. The endoplasmic reticulum serves a crucial role as an organelle within the cell. ER is essential for the regulation of protein translation, calcium storage, and lipid metabolism, which holds significant importance in the development of NAFLD and hepatocellular carcinoma [102]. The most apparent degenerative alterations manifested as dilated rough endoplasmic reticulum. Indeed, the endoplasmic reticulum is particularly susceptible to free radical attack, as it is recognized as a site of radical production. Consequently, the degenerative alterations in the endoplasmic reticulum may be attributed to heightened oxidative stress [103]. Endoplasmic reticulum stress may be crucial in the development of non-alcoholic fatty liver disease. Under stress conditions, the accumulation of unfolded proteins interacts with GRP78, leading to the release and subsequent activation of PERK, IRE1, and ATF6. The ER transmembrane proteins lead to the impairment of normal biological processes [104]. In our study, 16 weeks of ingestion of HFD resulted in dilation, swelling, and fragmentation of ER, attributed to ER stress, which leads to the generation of ROS and, furthermore, oxidative stress. El-khair et al. suggested that 12 weeks of treatment with HFD displayed ER stress and inflammation, which play an important role in NAFLD [35]. The present study displayed that supplementation with naringin alleviated the harmful effects of a high-fat diet. Ultrastructural examinations in the THN group suggested prominent euchromatin nuclei with distinct nucleolus, orderly distribution of ER, normal shape and size of mitochondria, no vacuolation of cytoplasm, and reduction of lipid droplets in comparison with the HFD group. EM study revealed that the presence of autophagosome and lysosome in the hepatocytes of the naringin treated group suggests naringin treatment promotes lipophagy and activates autophagic flux to alleviate NAFLD. All these findings indicate a reduction in ER stress and maintenance of ER homeostasis as well as attenuation of mitochondrial dysfunction. Moreover, naringin mitigates reticular stress associated with oxidative stress. Another study is accordance with our recent findings, depicted that naringin administration mitigates hepatic steatosis by reducing apoptosis in hepatocytes and promoting lipophagy in experimental NAFLD-mice model [105].

**Fig. 12:**
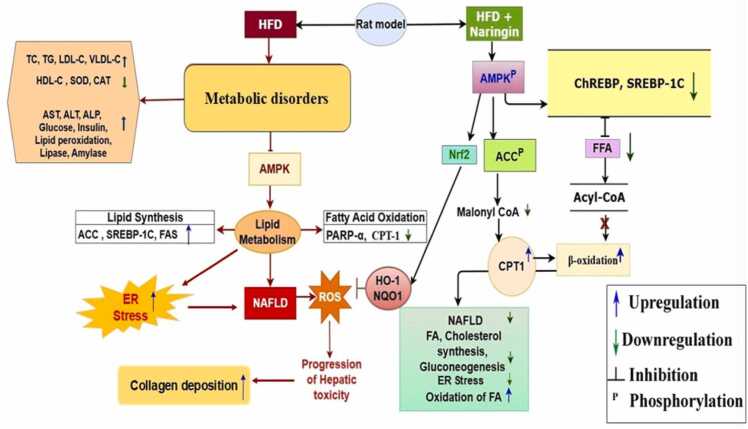
Mechanism of action of naringin to alleviate HFD-induced dyslipidemia, NAFLD and ER stress *via* downregulating ACC, FAS and SREBP-1C gene and upregulating AMPK gene, as well as inhibiting ROS generation by Nrf-HO-1/NQO-1 pathways. NAFLD: Non-alcoholic fatty liver disease; ER stress: Endoplasmic reticulum stress; FA: Fatty acid; FFA: Free fatty acid; FAS: Fatty acid synthase; ACC: Acetyl-coA Carboxylase; CPT-1: Carnitine palmitoyltransferase I; SREBP-1C: Sterol regulatory element binding protein-1C; AMPK: AMP-activated protein kinase; PARP-α: Poly (ADP-ribose) polymerase-α; ChREBP: Carbohydrate response element binding protein; HO-1: Heme oxygenase-1; NQO-1: NADPH quinone oxidoreductase; Nrf-2: Nuclear factor erythroid 2-related factor 2; TC: Total cholesterol; TG: Triglycerides; LDL-C: Low-density lipoprotein cholesterol; VLDL-C: Very low-density lipoprotein cholesterol; HDL-C: High-density lipoprotein cholesterol; AST: Aspartate aminotransferase; ALT: Alanine aminotransferase; ALP: Alkaline phosphatase.

In addition, this study contributes to the efficacy of the natural flavonoid naringin and its novelty to mitigate obesity and its related metabolic disorder, hepatotoxicity, as well as oxidative stress caused by the induction of a high-fat diet. In this study we also demonstrated the underlying mechanism of action (Fig. 12) of this phytochemical and its therapeutic approaches to combat dyslipidemia from the route via the AMPK and SREBP-1C gene interconnecting pathway. Further investigation should be needed for understanding the pharmacokinetics of naringin with other synthetic drugs and its underlying mechanism, which may give us new insight in the field of metabolic disorders.

## 5 Conclusion

The findings of the present study demonstrated that high-fat diet induced reticule stress in hepatic tissues which in turn resulted in NAFLD in experimental rats. Naringin (100 mg/kg B.W.) treatment mitigated reticule stress in liver tissues along with reducing fibrosis generation in hepatic tissues. For the first time, this study shows the efficacy of naringin to combat HFD-induced reticule stress in NAFLD conditions in experimental rats observed through transmission electron microscopic study. It could be concluded that naringin treatment might ameliorate liver tissue damage induced by high-fat diet by reducing ROS generation and maintaining lipid homeostasis, acting on AMPK and SREBP-1C genes. These findings may demonstrate new insight into the use of naringin as a potential therapeutic agent to combat obesity related hyperlipidemia and NAFLD which facilitates the adaptation of new dietary strategy to ameliorate high-fat diet-induced hepatotoxicity.

## CRediT authorship contribution statement

**Maharaj Biswas:** Validation, Supervision, Data curation, Conceptualization. **Sanjib Ghosh:** Writing – review & editing, Data curation. **Sweata Sarkar:** Writing – original draft, Methodology, Investigation, Funding acquisition, Formal analysis, Data curation, Conceptualization.

## Ethics approval and consent to participate

This investigation carried out in compliance with the guidelines for the care and use of laboratory animals. All experiments were conducted according to the guidelines set forth by the Committee for the Purpose of Control and Supervision of Experiments on Animals (Registration No: 892/GO/Re/S/01/CPCSEA), and received approval from the Institutional Animal Ethics Committee (IAEC), University of Kalyani.

## Funding

The current research did not obtain any particular funding from public, commercial, or charitable organisations.

## Declaration of Competing Interest

The authors declare that they have no known competing financial interests or personal relationships that could have appeared to influence the work reported in this paper.

## Data Availability

Data will be made available on request.
